# Radiosurgery and fractionated stereotactic body radiotherapy for patients with lung oligometastases

**DOI:** 10.1186/s12885-020-06892-4

**Published:** 2020-05-11

**Authors:** Goda G. Kalinauskaite, Ingeborg I. Tinhofer, Markus M. Kufeld, Anne A. Kluge, Arne A. Grün, Volker V. Budach, Carolin C. Senger, Carmen C. Stromberger

**Affiliations:** 1 Department of Radiation Oncology and Radiotherapy, Charité - Universitätsmedizin Berlin, Augustenburger Platz 1, 13353 Berlin, Germany; 2 Charité CyberKnife Center, Charité - Universitätsmedizin Berlin, Augustenburger Platz 1, 13353 Berlin, Germany; 3 The Translational Radiooncology and Radiobiology Research Laboratory, Charité - Universitätsmedizin Berlin, Berlin, Germany

**Keywords:** Oligometastases, SBRT, Radiosurgery, Lung metastases, CyberKnife

## Abstract

**Background:**

Patients with oligometastatic disease can potentially be cured by using an ablative therapy for all active lesions. Stereotactic body radiotherapy (SBRT) is a non-invasive treatment option that lately proved to be as effective and safe as surgery in treating lung metastases (LM). However, it is not clear which patients benefit most and what are the most suitable fractionation regimens. The aim of this study was to analyze treatment outcomes after single fraction radiosurgery (SFRS) and fractionated SBRT (fSBRT) in patients with lung oligometastases and identify prognostic clinical features for better survival outcomes.

**Methods:**

Fifty-two patients with 94 LM treated with SFRS or fSBRT between 2010 and 2016 were analyzed. The characteristics of primary tumor, LM, treatment, toxicity profiles and outcomes were assessed. Kaplan-Meier and Cox regression analyses were used for estimation of local control (LC), overall survival (OS) and progression-free survival.

**Results:**

Ninety-four LM in 52 patients were treated using SFRS/fSBRT with a median of 2 lesions per patient (range: 1–5). The median planning target volume (PTV)-encompassing dose for SFRS was 24 Gy (range: 17–26) compared to 45 Gy (range: 20–60) in 2–12 fractions with fSBRT. The median follow-up time was 21 months (range: 3–68). LC rates at 1 and 2 years for SFSR vs. fSBRT were 89 and 83% vs. 75 and 59%, respectively (*p* = 0.026). LM treated with SFSR were significantly smaller (*p* = 0.001). The 1 and 2-year OS rates for all patients were 84 and 71%, respectively. In univariate analysis treatment with SFRS, an interval of ≥12 months between diagnosis of LM and treatment, non-colorectal cancer histology and BED < 100 Gy were significantly associated with better LC. However, none of these parameters remained significant in the multivariate Cox regression model. OS was significantly better in patients with negative lymph nodes (N0), Karnofsky performance status (KPS) > 70% and time to first metastasis ≥12 months. There was no grade 3 acute or late toxicity.

**Conclusions:**

Longer time to first metastasis, good KPS and N0 predicted better OS. Good LC and low toxicity rates were achieved after short SBRT schedules.

## Background

Metastatic progression of cancer is linked to poor prognosis and is the leading cause of cancer-related deaths [1]. Few decades ago, the diagnosis of metastatic disease was related to lethal outcomes. This paradigm has changed after Hellman and Weichselbaum introduced the concept of oligometastases: the intermediate state between non-metastatic cancer and highly palliative disseminated metastatic disease [2]. Patients with an initially limited number of metastases or with progression of only few lesions after cytoreductive therapy might be potentially cured or reach long-term survival when treated with local ablation therapy for all lesions. The search for prognostic biomarkers for discrimination of potentially oligometastatic patients is still ongoing. In some small prospective studies circulating tumor cells as well as circulating tumor DNA in liquid biopsies were able to predict treatment outcomes and response to ablative therapy [3]. However, until prognostic biomarkers will be established for routine application, the selection of patients that could benefit from local ablative therapy rather than from palliation will be based on clinical features.

The lungs are one of the most common metastatic sites for various solid tumors [4, 5]. Stereotactic body radiotherapy (SBRT) and surgical resection are frequently used treatment options for patients with a limited number of pulmonary lesions. Although SBRT compared to surgery for lung metastases have not been studied in a prospective randomized trial, retrospective data suggest that both methods achieve equal results in terms of local control and overall survival [6, 7]. Single fraction radiosurgery (SFRS) is especially attractive as an outpatient procedure in terms of patients’ compliance, cost effectiveness and limited treatment time. However, up to now there is no recommendation when to administer SFRS over fractionated SBRT (fSBRT). The aim of this study was to analyze local control (LC) after SFRS and fSBRT in patients with lung oligometastases and identify prognostic clinical features for better survival outcomes.

## Methods

### Study design

This retrospective study was approved by the institutional medical ethics committee of the Charité - Universitätsmedizin Berlin (EA1/214/16). We identified all patients with lung metastases treated with curative intended SFRS or fSBRT between January 2010 and December 2016. Cases with an initially limited number of lung metastases from various solid tumors or with oligo-progression after systemic therapy were selected for the study. Patients with disseminated disease or with a second malignancy were excluded. The data on patients’ demographics, e.g. primary tumor and metastases, disease stage as determined by computed tomography (CT), magnetic resonance imaging or positron emission tomography, treatment parameters, follow-up and LC, overall survival (OS), progression-free survival (PFS), distant metastases-free survival (DMFS) were calculated. Clinical follow-up was performed at 6 weeks after SFRS/fSBRT and at 3, 6, 12, 18, and 24 months after treatment and annually thereafter. Acute and late adverse events were scored using NCI Common Terminology Criteria for Adverse Events (CTCAE), version 4.0.

### Treatment planning and delivery

SBRT was delivered using CyberKnife (CK) and Novalis systems, both dedicated stereotactic linear accelerators. For respiratory motion compensation, the CyberKnife Synchrony® Respiratory Motion Tracking System was used. In general, one gold fiducial (1.0 mm × 5.0 mm) was placed centrally within the lung metastasis under CT-guidance in local anesthesia. For lesions larger than 2 cm feasibility of X-sight lung tracking was evaluated. If motion compensation was not possible (e.g. due to patients’ comorbidities or technical limitations) an internal gross tumor volume (IGTV), defined as the gross tumor volumes of all respiratory phases on a 4D CT was constructed. In these cases, patients were aligned on the spine. High-resolution thin-slice native planning CT of the chest with 1.0 to 2.0 mm slice thickness in supine position was performed.

The gross tumor volume (GTV) was delineated on all axial slices including spiculae in the lung window. The clinical target volume (CTV) was equal to the GTV. The planning target volume (PTV) was obtained by adding a 5–8 mm margin to the CTV.

**Fig. 1:**
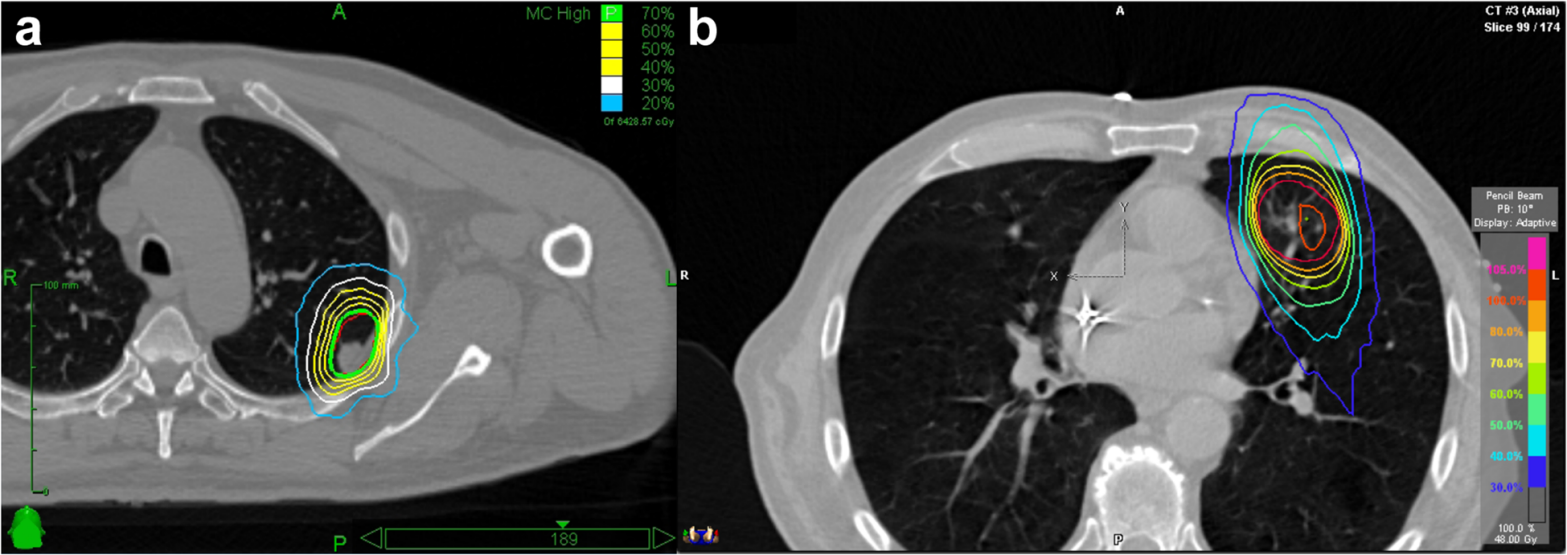
Treatment plan and dose distribution for (**a)** CyberKnife, (**b**) Novalis treatment system

For CK treatments, doses were prescribed to the 70% isodose covering the PTV and a total maximum of 100%. Novalis treatment was planned with less inhomogeneous dose distributions with the 80% isodose line of the prescribed 100% dose encompassing the PTV and allowing a maximum of up to 110% (Fig. 1).

The linear-quadratic model, assuming an alpha/beta ratio of 10 Gy for tumor, was used to calculate the biologically equivalent dose (BED) and the equivalent dose in 2 Gy fractions (EQD2) for PTV-encompassing total dose. Dose constraints to organs at risk for single fraction treatment are shown in Table 1. Treatment planning for CK was performed in Multiplan® (Accuray) using the Ray-Trace or Monte Carlo algorithm and for Novalis in iPlan® (BrainLAB) using the Pencil Beam algorithm.

**Table 1:** Dose constrains for organs at risk of single fraction radiosurgery

Organs at risk	Max critical volume above threshold (cm^3^)	Threshold dose (Gy)	Max point dose (Gy)^a^
Spinal cord	<0.35	10.0	14.0
Esophagus	<5	11.9	15.4
Hearts/pericardium	<15	16.0	22.0
Great vessels	<10	31.0	37.0
Trachea and large bronchus	<4	10.5	20.2
Rib	<1	22.0	30.0
Ipsilateral Lung (mean)	-	9.0	-

^a^Point defined as 0.035 cm^3^ or less

### Endpoints and statistical considerations

LC was defined as time from SFRS/fSBRT to tumor progression within the irradiation field or absence of progression at last available follow-up. LC was assessed using routinely CT scans every 3 months. PET-CT and/or biopsy of irradiated metastasis was performed in cases of uncertain progression detected on CT images. OS was calculated from the beginning of SFRS or fSBRT until the death of any cause or the date of last follow-up. The time to new metastases in the lung outside of the SFRS/fSBRT field or in other organs was defined as DMFS and was calculated from the start of SFRS/fSBRT. PFS was defined as the time from the start of SFRS/fSBRT until progression of the primary tumor, development of new metastases or local failure.

LC was compared between lung metastases treated with SFRS and fSBRT. The different fractionation regimens in the same patient were allowed, thus fractionation impact on OS, PFS and DMFS could not be assessed.

OS, LC, DMFS and PFS after SFRS/fSBRT for lung metastases were calculated using the Kaplan-Meier method. Cox-regression analysis was used to obtain the Hazard Ratio (HR) and 95% confidence intervals (CI) for various covariates. Covariates with a *p*-value of ≤0.1 were included into the multivariate analyses carried out with a Cox proportional hazards model with a threshold of *p* < 0.05. The chi-squared test was performed in order to compare variables between groups. A p-value of < 0.05 was considered as statistically significant. The data processing and statistical analyses were accomplished using FileMaker Pro 15 Advanced, Excel 2010 and IBM SPSS Statistics 24 (SPSS Inc., Chicago, IL, USA).

## Results

### Patient and tumor characteristics

The clinical, treatment and follow-up data of 52 eligible patients were assessed. Thirty-two patients were male (61.5%) and 20 were female (38.5%) with a median age of 66 years (range: 26–84) and a median Karnofsky performance status (KPS) of 80% (range: 60–100). The most prevalent primary tumor was colorectal cancer (CRC) in 17 patients (32.7%). PET-CT staging before the SBRT for lungs was performed in 7 (13.5%) patients. Twelve patients (23.1%) had oligometastases at the time of tumor diagnosis. The median time to first metastasis was 19.5 months (range: 0–37.9). In 37 patients (71.2%) metastases were limited to the lungs. Eight patients (15.4%) had additional liver metastases and 3 patients (5.8%) had brain metastasis. Forty-six patients (88.5%) had systemic therapy prior to lung SBRT and 15 (28.8%) after lung SBRT. Seventeen patients (32.7%) received immunotherapy at any time during the disease course. Patients’ and primary tumor characteristics are shown in Table 2.

**Table 2:** Patient and primary tumor characteristics

Characteristics	No. (%)
Age, years	
Median	66
Range	26 - 84
Gender	
Female	20 (38.5)
Male	32 (61.5)
KPS (%)	
Median	80
Range	60 - 100
Primary tumor type	
CRC	17 (32.7)
Sarcoma	8 (15.4)
Melanoma	7 (13.5)
HNC	6 (11.5)
RCC	5 (9.6)
NSCLC	3 (5.8)
Others	6 (11.5)
T-classification at initial diagnosis	
T≤2	17 (32.7)
T>2	30 (57.7)
Unknown	5 (9.6)
N-classification at initial diagnosis	
N0	18 (34.6)
N+	26 (50.0)
Unknown	8 (15.4)
M-classification at initial diagnosis	
M0	36 (69.2)
M1	12 (23.1)
Unknown	4 (7.7)
Pre-SFRS/fSBRT systemic therapy	
Yes	46 (88.5)
No	6 (11.5)
No. of LM treated with SFRS/fSBRT per patient	
Median	2
Range	1 - 5
No. of affected organs per patient	
Median	1
Range	1 - 4

*KPS* Karnofsky performance status*, CRC* colorectal cancer, *HNC* head and neck cancer, *RCC* renal cell carcinoma, *NSCLC* non-small cell cancer, *SFRS* single fraction radiosurgery, *fSBRT* fractionated stereotactic body radiotherapy, *LM* lung metastasis

### Treatment characteristics

Overall, 94 lung metastases were treated using SFRS/fSBRT with a median of 2 lesions per patient (range: 1–5). Metastases and SFRS/fSBRT characteristics are shown in Table 3 and Table 4. Forty-five metastases (47.9%) were treated with SFRS of which only 12 were located centrally. Metastases treated with fSBRT were almost equally distributed with respect to location (24 central vs. 25 peripheral). Median diameter of metastases was 14.5 mm (range: 5–70), with no significant difference between centrally and peripheral located lesions. The median time from the diagnosis of lung metastases to the start of SFRS/fSBRT was 4.5 months (range: 0–61). Before the therapy with CK a gold fiducial was implanted in 51 metastases, whereof 37 were treated with SFRS and 14 with fSBRT using the Synchrony tracking method. A total of 14 lung metastases were treated using the X-sight lung tracking method. IGTV was used for all 29 metastases treated with Novalis. The median prescription dose for SFRS was 24 Gy (range: 17–26) compared to fSBRT with median 45 Gy (range: 20–60) delivered in 2–12 fractions. The median diameter and PTV were significantly smaller in metastases treated with SFRS compared to fSBRT: 12 mm (range: 5–35) and 9.9 cm^3^ (range: 2.4–90.8) vs. 16 mm (range: 5–70) and 24.0 cm^3^ (range: 5.8–164.5), respectively.

**Table 3:** Metastases and treatment characteristics

LM and treatment characteristics	SFRS (*n*=45)	fSBRT (*n*=49)	*p*-value
Metastasis diameter (mm)			
Median	12.0	16.0	0.003
Range	5.0-35.0	5.0-70.0	
Metastasis PTV (cm^3^)			
Median	9.9	24.0	<0.001
Range	2.4-90.8	5.8-164.5	
Metastasis location			
peripheral	32	25	0.092
central	13	24	
Metastasis histology (CRC vs. non-CRC)			
CRC	8	21	0.009
Non-CRC	37	28	
PTV-encompassing prescription dose (Gy)			
Median	24	45	<0.001
Range	17-26	20-60	
PTV-encompassing single dose (Gy)			
Median	24	9.6	<0.001
Range	17-26	4-16	
Biological effective dose (Gy)			
Median	81.6	105.6	0.015
Range	45.9-93.6	42.6 – 151.2	

*LM* lung metastases*, SFRS* single fraction radiosurgery, *fSBRT* fractionated stereotactic body radiotherapy, PTV planning target volume, *CRC* colorectal cancer

**Table 4:** Fractionation regimens

Fractions and PTV- encompassing single dose	No. of LM (%)	BED (Gy)	EQD2 (Gy)
1 x 22 Gy	2 (2.1)	70.4	58.7
1 x 24 Gy	20 (21.3)	81.6	68.0
1 x 25 Gy	12 (12.8)	87.5	72.9
1 x 26 Gy	5 (5.3)	93.6	78.0
3 x 12.5 Gy	3 (3.2)	84.4	70.3
3 x 15 Gy	8 (8.5)	112.5	93.8
3 x 16 Gy	9 (9.6)	124.8	104.0
4 x 12 Gy	8 (8.5)	105.6	88.0
4 x 9.6 Gy	9 (9.6)	75.3	62.7
5 x 8 Gy	2 (2.1)	72.0	60.0
other regimens	16 (17.0)		

*LM* lung metastases*, PTV* planning target volume*, BED* biologically effective dose*, EQD2* equivalent dose

### Patient outcomes

**Fig. 2:**
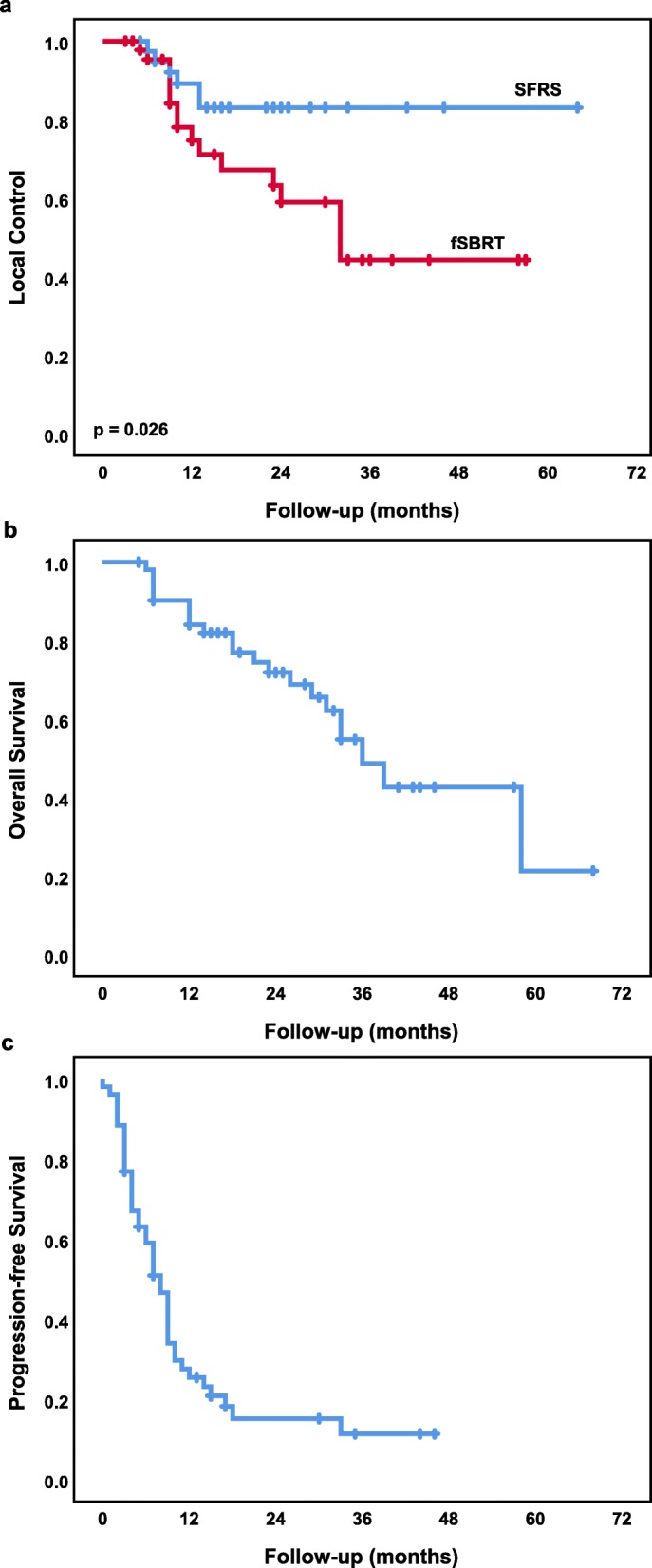
Kaplan-Meier curves of (**a**) local control SFRS vs. fSBRT, (**b)** overall survival, (**c**) progression-free survival

The median follow-up time was 21 months (range: 3–68). The 1-year and 2-year LC rates for SFSR vs. fSBRT were 89 and 83% vs. 75 and 59%, respectively (*p* = 0.026). One and 2-year LC rates for metastases from CRC vs. non-CRC were 59 and 46% vs. 90 and 80%, respectively (*p* = 0.001). In 5 out of 22 metastases with local progression relapse was confirmed using PET-CT and in 2 after histological examination. Eleven lesions were repeatedly treated with local therapy: either with repeated SBRT or with surgery. One and 2-year OS and PFS rates were 84, 71 and 26%, 15%, respectively. At the time of analysis 21 patients (41.4%) were dead. Disease progression occurred in 42 patients (80.8%), of which 19 patients (36.5%) developed metastases in new organs. The Kaplan-Meier LC, OS and PFS curves are shown in Fig. 2.

Treatment with SFRS, an interval of < 12 months between diagnosis of metastases and the beginning of SFRS/fSBRT as well as non-colorectal histology were significantly associated with better LC in univariate analysis (Table 5). However, none of these parameters remained significant in multivariate analysis. N0, KPS > 70% and time to first metastasis ≥12 months were significantly associated with improved OS. PFS was significantly better in patients with KPS > 70% and with maximum 3 metastases at the time of SBRT (Table 6). There was no difference regarding survival outcomes between patients with oligorecurence and oligometastases.

**Table 5:** Univariate analysis of factors influencing local control

Covariate	HR (95% CI)	*p*-value
Time between diagnosis of LM and SBRT (months)		
<12	1	
≥12	2.5 (1.1-6.0)	0.027
Location of LM		
central	1	
peripheral	0.7 (0.2-1.7)	0.412
Histology		
CRC	1	
non-CRC	0.2 (0.1-0.6)	0.004
LM diameter (mm)		
≤10	1	
>10	2.2 (0.8-6.6)	0.150
PTV (cm^3^)		
≤10	1	
>10	3.3 (0.9-11.3)	0.053
Fractionation regimens		
SFRS	1	
fSBRT	2.7 (1.0-7.0)	0.037
BED		
<100Gy	1	
≥100 Gy	2.7 (1.1-6.4)	0.021

*HR* Hazard ratio, *CI* confidence interval, *LM.* lung metastases, *SBRT* stereotactic body radiotherapy, *SFRS* single fraction radiosurgery, *fSBRT* fractionated stereotactic body radiotherapy, PTV Planning target volume, *BED* biologically effective dose

**Table 6:** Univariate and multivariate analysis of factors influencing overall and progression-free survival

Covariate	Overall survival				Progression-free survival			
	Univariate analysis	Multivariate analysis			Univariate analysis		Multivariate analysis	
	HR (95% CI)	*p*-value	HR (95% CI)	*p*-value	HR (95% CI)	*p*-value	HR (95% CI)	*p*-value
Age (years)								
>70	1				1			
≤70	1.1 (0.4-2.7)	0.81	NA	NA	0.8 (0.4-1.5)	0.56	NA	NA
Gender								
Female	1				1			
Male	1.6 (0.6-4.6)	0.31	NA	NA	1.2 (0.8-1.6)	0.25	NA	NA
Primary tumor								
non-CRC	1				1			
CRC	0.6 (0.2-1.4)	0.29	NA	NA	0.8 (0.5-1.6)	0.64	NA	NA
KPS								
≤70%	1				1			
>70%	0.4 (0.2-1.1)	0.09	0.3 (0.1-0.8)	0.03	0.5 (0.3-0.9)	0.03	0.4 (0.2-0.7)	0.02
T-classification								
T≤2	1				1			
T>2	2.4 (0.8-6.8)	0.08	1.5 (0.4-5.0)	0.48	1.4 (0.7-2.8)	0.31	NA	NA
N-classification								
N0	1				1			
N+	2.6 (0.9-7.3)	0.06	4.4 (1.2-15.6)	0.02	1.4 (0.7-2.7)	0.33	NA	NA
Time to first metastasis (months)								
<12	1				1			
≥12	0.3 (0.1-0.9)	0.03	0.2 (0.1-0.7)	0.01	0.6 (0.3-1.2)	0.14	NA	NA
No. of metastases before SBRT								
<3	1				1			
≥3	1.4 (0.6-3.3)	0.42	NA	NA	2.6 (1.3-5.1)	0.005	2.7 (1.4-5.4)	0.003
No. of affected organs								
1	1				1			
>1	1.6 (0.7-3.9)	0.24	NA	NA	1.1 (0.5-1.9)	0.97	NA	NA
Systemic therapy before SBRT								
Yes	1				1			
No	1.4 (0.3-6.3)	0.65	NA	NA	1.4 (0.5-4.1)	0.48	NA	NA

*NA* not assessed, *HR* Hazard ratio, *CI* confidence interval, CRC colorectal cancer, *KPS* Karnofsky performance status, *SBRT* stereotactic body radiotherapy

### Treatment related toxicity

The SFRS and fSBRT were safe and very well tolerated. No treatment-related deaths and grade ≥ 3 toxicities occurred. Six patients (11.5%) developed asymptomatic grade 1 pneumonitis (2 patients after SFRS and 4 patients after fSBRT) and one patient had grade 1 pulmonary fibrosis. Symptomatic and medical intervention requiring grade 2 pneumonitis was diagnosed in one patient (1.9%) after SFRS with 25 Gy.

## Discussion

This analysis represents a single-center experience in treating oligometastatic lung lesions with curative intended SFRS and fSBRT. The 1-, 2-year LC and OS rates for the entire cohort were 82, 70 and 84%, 71%, respectively. Our findings are comparable with the current findings in the literature (Table 7) [8–16].

**Table 7:** Overall survival and local control rates after SFRS/fSBRT or pulmonary metastasectomy according to various studies

Reference	Study design	Year	No. Patients	Primary tumor	No. of LM	Treatment	Overall survival		Local control	
							1-year (%)	2-years (%)	1-year (%)	2-years (%)
Nuyttens et al. [8]	Phase 2 study	2015	30	Various	1 - 5	SFRS/fSBRT	-	63	79	-
Rieber J et al. [9]	Retrospective	2016	700	Various	42% single	SFRS/fSBRT	75.1	54.4	-	81.2
Navarria et al. [10]	Retrospective	2014	76	Various	1 - 5	fSBRT	84.1	73	95	89
Sharma A. et al. [11, 12]	Retrospective	2018	206	Various	1 - 5	SFRS/fSBRT	-	63	-	85
Widder J et al. [13]	Retrospective	2013	110	Various	3 - 5	fSBRT 42, PME 68	SBRT: 87 PME: 98	SBRT: 86 PME: 74	SBRT: 94 PME: 93	SBRT:94 PME:90
Sapir et al. [14]	Retrospective	2016	78	Sarcoma	-	SBRT 26, PME 127	-	SBRT: 57.9, PME: 62.2	-	SBRT: 97.4 PME: 96.8
Filippi et al. [15]	Retrospective	2014	67	Various	1 - 5	SFRS	85.1	70.5	93	88.1
Agolli L [16]	Retrospective	2017	44	CRC	1 - 4 (61% single)	SFRS/fSBRT	-	67.7	68.8	60.2
Present study	Retrospective	2019	52	Various	Median 2	SFRS/fSBRT	84	71	SFRS 89, fSBRT 83	SFRS 83, fSBRT 59

*LM* lung metastases*, SBRT* stereotactic body radiotherapy, *SFRS* single fraction radiosurgery, *fSBRT* fractionated stereotactic radiotherapy

SBRT is an attractive non-invasive treatment option providing good therapy outcomes with minimum toxicity. The BED ≥100 Gy, smaller tumor size, shorter interval between diagnosis and treatment of metastases are favorable prognostic factors influencing local control of lung metastases after SBRT [9, 17–19]. The existing data on fractionation schedules as well as dosage of SBRT for lung metastases is limited by retrospective nature or non-randomized prospective study design. Therefore, no standardized treatment regimens are yet available. The primary results of TROG 13.01 SAFRON II Phase II trial which compares SFRS to fSBRT for lung metastases are expected soon [20].

According to our data, small lung metastases (median PTV ≤ 9.9 cm^3^, median diameter 12 mm) might safely be treated with SFRS applying 24–26 Gy (median D_max_ of 53 Gy and a median BED_max_ of 81 Gy) with excellent 1- and 2-year LC rates of 89 and 83%, implying that BED < 100 Gy using SFRS might be sufficient for durable control in small lung lesions. This observation, however, contradicts the findings of other studies, where BED < 100 Gy was found to be a negative prognostic factor for LC. Ricco et al. analyzed whether different lung metastases volumes and BED were associated with treatment outcomes [17]. In this study, lesions after SBRT with BED ≥100 Gy reached better LC rates. Moreover, in the group with BED ≥100 Gy smaller metastases (volume < 11 cm^3^) were linked to improved LC and OS rates. The median number of fractions employed was 3 (range: 1–8), how many lesions were treated with SFRS remains unclear. Other trials rarely report on the significance of BED and fractionation regimens in terms of treatment outcome for metastases according to their size [9, 12]. Nevertheless, the existing data on size-adapted SFRS for lung metastases as well as primary lung tumors is promising with 1 year LC rates varying from 89.1–93.4% [15, 21–23]. However, diverse measurement units or target volumes describing metastases size (e.g. diameter, GTV, PTV) found in the literature make it difficult to categorize lesions or to identify the optimal dose. Randomized, prospective studies are needed to determine which fractionation schedule is the most suitable for lung metastases according to the size in terms of therapy outcomes, toxicity and patient’s compliance.

In the current study, 1- and 2-year LC rates for metastases from CRC compared with non-CRC were significantly worse. Recently, Jingu et al. investigated the impact of primary tumor histology on LC rates after SBRT for lung metastases in a metanalysis and systematic review. Analysis of 1920 patients (619 with CRC, 1301 non-CRC) showed that LC was significantly inferior in the CRC group (*p* < 0.00001). In addition, the dose escalation (BED > 130 Gy) was associated with decreased local recurrences [24]. Furthermore, Ahmed and colleagues concluded that lung metastases from rectal carcinoma are related with increased radio-resistance, and therefore are more likely to relapse after SBRT. The authors recommend dose escalation with BED > 100 Gy for radio-resistant tumors in order to improve treatment outcomes [25]. In the present study, the median BED for relapsed metastases from rectal cancer was 87.5 Gy (range: 56–124.8), suggesting that an insufficient dose for this histology may be responsible for lower LC rates in patients with CRC. Therefore, SBRT with BED < 100 Gy should be used with caution in patients with lung oligometastases from rectal cancer.

We found time to the first metastasis ≥12 months, KPS > 70% and N0 to be independent favorable prognostic factors for OS. Metachronous metastases with longer metastasis free interval are associated with indolent tumor histology and thus are frequently linked to better outcomes, with the favoring time to metastasis diagnose varying from ≥2 months to ≥75 months depending on the primary tumor type [26–28]. Furthermore, in agreement with our results good performance score before initiation of the SBRT was linked to better survival in various studies [29, 30]. Absence of lymph node involvement was addressed as a prognostic factor mostly in series on oligometastatic lung cancer [27, 31]. Unlike our finding, no prognostic value of N classification was reported in studies with cohorts of heterogenous primary tumor type, therefore this finding must be interpreted carefully. Despite the small sample size, we identified two commonly reported prognostic factors that might be useful for selecting oligometastatic patients for curative SBRT.

The major limitation of this study is its retrospective design with inhomogeneous primary tumor types and the limited number of patients. Therefore, neither a subgroup analysis based on metastasis histology nor an analysis of the effects of dose escalation was performed. Treatment planning calculations with Ray-Tracing, Pencil Beam or Monte Carlo dose algorithms for lung might produce differences in dose distribution for target and organs at risk. However, there was no difference detected in the treatment outcomes in metastases planed with different treatment algorithms. Since multiple metastases in the same patient were treated with different fractionation, finding the prognostic value of SFRS vs. fSBRT for survival outcomes was not feasible.

## Conclusions

KPS > 70%, longer time to first metastasis and absence of locoregional lymph node metastases were found to be positive predictive factors for OS in patients with lung oligometastases after SBRT. Long-term LC and low toxicity rates were achieved after short SBRT schedules.

## Abbreviations

BED: Biologically effective dose
CRC: Colorectal cancer
CI: Confidence interval
CT: Computed tomography
CTV: Clinical treatment volume
CK: Cyberknife
DMFS: Distant metastases-free survival
EQD2: Equivalent dose in 2 Gy fractions
fSBRT: Fractionated stereotactic body radiotherapy
GTV: Gross tumor volume
HNC: Head and neck cancer
HI: Hazard ratio
IGTV: Internal gross tumor volume
LC: Local control
non-CRC: Non-colorectal cancer
NSCLC: Non-small-cell lung cancer
OS: Overall survival
PFS: Progression-free survival
PTV: Planning treatment volume
RCC: Renal cell carcinoma
SFRS: Single fraction radiosurgery
SBRT: Stereotactic body radiotherapy
